# Screening and identification of autophagy‐related biomarkers for oral squamous cell carcinoma (OSCC) via integrated bioinformatics analysis

**DOI:** 10.1111/jcmm.16512

**Published:** 2021-04-09

**Authors:** Guang‐zhao Huang, Zhi‐yun Lu, Yu Rao, Hai Gao, Xiao‐zhi Lv

**Affiliations:** ^1^ Department of Oral & Maxillofacial Surgery NanFang Hospital Southern Medical University Guangzhou China; ^2^ Department of Prosthodontics Stomatological Hospital Southern Medical University Guangzhou China

**Keywords:** ATG12, autophagy, BID, bioinformatics, oral squamous cell carcinoma, prognosis

## Abstract

Increasing evidences have showed that autophagy played a significant role in oral squamous cell carcinoma (OSCC). Purpose of our study was to explore the prognostic value of autophagy‐related genes (ATGs) and screen autophagy‐related biomarkers for OSCC. RNA‐seq and clinical data were downloaded from The Cancer Genome Atlas (TCGA) database following extracting ATG expression profiles. Then, differentially expressed analysis was performed in R software and a risk score model according to ATGs was established. Moreover, comprehensive bioinformatics analyses were used to screen autophagy‐related biomarkers which were later verified in OSCC tissues and cell lines. A total of 232 ATGs were extracted, and 37 genes were differentially expressed in OSCC. Gene Ontology (GO) and Kyoto Encyclopedia of Genes and Genomes (KEGG) analysis demonstrated that these genes were mainly located in autophagosome membrane and associated with autophagy. Furthermore, the risk score on basis of ATGs was identified as potential independent prognostic biomarker. Moreover, ATG12 and BID were identified as potential autophagy‐related biomarkers of OSCC. This study successfully constructed a risk model, and the risk score could predict the prognosis of OSCC patients accurately. Moreover, ATG12 and BID were identified as two potential independent prognostic autophagy‐related biomarkers and might provide new OSCC therapeutic targets.

## 1 INTRODUCTION

Oral squamous cell carcinoma (OSCC) is one of the most common head and neck squamous cell carcinoma in the worldwide.^1^ Smoking, drinking, betel nut chewing and human papillomavirus (HPV) infection are the most common risk factors.^2^ Despite the advance in medical equipment and treatment methods, the overall survival rate of OSCC is still unsatisfied. It is mainly owing to lack of effective biomarkers for early‐stage diagnosis and accurate prediction of local recurrence and distant metastasis.^3^ Therefore, it is crucial to identify the effective biomarkers and therapeutic targets for OSCC to improve the overall survival.

Autophagy is characterized by sequestration of bulk cytoplasm, long‐lived proteins and damaged cellular organelles encapsulated as autophagosomes and delivered for lysosomal degradation to recycle the nutrients, and it is a cellular self‐consumption process. Autophagy is regulated by the conversion of the cytoplasmic microtubule‐associated protein 1 light chain 3 (LC3‐I) into the membrane form of protein 2 light chain 3(LC3‐II) which promotes the autophagosomal degradation.^4^ In summary, autophagy is a lysosomal‐mediated catabolic complex process which involves the cytoplasmic organelles and proteins to maintain metabolism and homeostasis in cells.^5^ Recently, numerous studies have showed that autophagy dysregulation played significant roles in a variety of human malignancies, including colorectal cancer,^6^ renal cell carcinoma,^7^ non‐small cell lung cancer^8^ and so on. In addition, majority of evidences have showed that autophagy might play significant roles in OSCC carcinogenesis. For example, autophagy‐mediated cell apoptosis to promote tumour progression via the AKT/mTOR pathway in OSCC.^9^ Dysregulation of autophagy process was relevant to tumorigenesis and prognosis in OSCC.^10^ Therefore, bioinformatics analysis of ATGs may reveal its prognostic value and provide potential therapeutic targets for OSCC treatment.

The purpose of this study was to analyse the differentially expressed ATGs in OSCC and establish a cox regression model to predict the overall survival of OSCC patients. Furthermore, survival analyses combined with stratification analyses were performed to identify the accuracy of cox formula. The present study may provide a novel insight into the potential mechanisms in OSCC initiation and progression and new therapeutic targets for OSCC treatment.

## 2 MATERIALS AND METHODS

### 2.1 Data downloading

The RNA‐seq data of 351 cases were downloaded from The Cancer Genome Atlas (TCGA) database (https://portal.gdc.cancer.gov/). Owing to its half‐baked clinical data, two samples were excluded. Finally, 317 OSCC samples and 32 normal controls were enrolled in our study. Subsequently, a total of 232 ATGs were extracted from transcriptome profiles in R software (Version 3.6.1). Then, differentially expressed analysis was performed in R software EdgeR package with the cut‐off criteria |log_2_ (fold change [FC])|>1.0 and FDR (adjusted P‐value)<.01.

### 2.2 GO and KEGG analysis

Gene Ontology (GO) analysis was performed to analyse these differentially expressed ATGs in DAVID database (https://david.ncifcrf.gov/). In addition, the Kyoto Encyclopedia of Genes and Genomes (KEGG) pathway enrichment analysis was employed to annotate the functions. The significance level of *P* < .05 was taken as the cut‐off standard.

### 2.3 Construction of cox regression model

The ATG profiles were transformed and normalized in a log_2_(x + 1) manner.^11^ Firstly, all ATGs were enrolled in univariate Cox regression to screen prognostic genes with *P*‐value < 0.05. Then, stepwise regression analysis was used to construct the Cox risk model according to these prognostic genes. Finally, 9 ATGs were enrolled in risk Cox regression, and the visualizations of Cox model were performed in R software according to the risk score for each patient. In addition, the predictive power of the signature was evaluated using the receiver operating characteristic (ROC). And univariate and multivariate cox regression analyses were used to analyse the clinical characteristics including age, gender, grade, stage, T, N stage in TNM system and risk score. M classification was eliminated because of lots of missing data.

### 2.4 Survival analysis based on cox model

On basis of cox model, OSCC patients in TCGA were segmented into high‐ and low‐risk level group. Survival analysis and stratification analysis including age, gender, grade, stage, T and N classification in TNM system along with a log rank p test were applied to validate its accuracy in R software survival package.

### 2.5 Collection of OSCC specimens

50 cases of OSCC tissues and matched normal mucosa tissues (MNTs) were collected from Nanfang Hospital, Southern Medical University. MNTs at least 1.5 cm from the edge of the tumour were defined as normal controls. All tumour tissues and MNTs were, respectively, confirmed as squamous cell carcinoma and normal tissues pathologically. According to median value of ATG12 and BID mRNA level, OSCC samples were divided into high‐ and low‐expression group. Subsequently, correlation analysis between ATG12 and BID expression level and clinical characteristics was performed.

### 2.6 Cell culture

OSCC cell lines (SCC9, SCC15, SCC25) and Human Oral Keratinocytes (HOK) were obtained from Institute of Antibody Engineering, Southern Medical University. Cell lines HOK, SCC15 and SCC25 were seeded in DMEM (*Gibco, Cat#11995500TB*) and SCC9 in DMEM/F12 (*Gibco, Cat#C11330500BT*) containing 10% foetal bovine serum(FBS) (*ExCell Bio, Inc*) and incubated at 37°C with 5% CO_2_.

### 2.7 RNA extraction and quantitative real‐time PCR (qRT‐PCR)

Total RNAs of tissues and OSCC cells were extracted with TRIzol reagent (*Takara, Cat# 9109*), and the same amount of total RNAs were reversed to cDNA according to the Reverse Transcription Kit manufacturer's protocol (*Vazyme*). Expression level of crucial ATGs was detected by qRT‐PCR with ChamQ Universal SYBR qPCR Master Mix (*Vazyme Biotech Co., Ltd*) and normalized to GAPDH. The sequences of the PCR primers were as follows: ATG12, forward 5′‐CTGCTGGCGACACCAAGAAA‐ 3′ and reverse 5′‐CGTGTTCGCTCTACTGCCC‐3′; BID, forward 5′‐ATGGACCGTAGCATCC CTCC‐3′ and reverse 5′‐GTAGGTGCGTAGGTTCTGGT‐ 3′; GAPDH, forward 5′‐CGCTGA GTACGTCGTGGAGTC‐3′ and reverse 5′‐GCTGATGATCTT GAGGCTGTTGTC‐3′.

### 2.8 Western blot

RIPA lysis buffer was used to extract OSCC cell lines’ protein. Furthermore, protein samples were separated, transferred to PVDF membranes (*Pall, Cat# BSP0161*) and then sealed with 5% skim milk. Subsequently, primary antibodies were incubated at 4℃ for overnight and second antibodies were incubated at room temperature for 1 hour. Finally, protein level was quantified by ECL (*YEASEN, Cat# 36208ES76*). The antibodies’ information was as follows. ATG12 (*ABclonal, Cat#A8033,1:1000*); BID (*Bioss, Cat#bs‐1153R,1:2000*);α‐tublin (*Proteintech, Cat# 66031‐1‐1g,1:5000*); goat anti‐rabbit (*Proteintech, Cat#SA00001,1:10 000*); and goat anti‐mouse (*Proteintech, Cat#SA00001‐1,1:10 000*).

### 2.9 Immunohistochemistry

OSCC tissues and MNTs were fixed with 4% formaldehyde, dehydration as well as wax immersion, embedded in paraffin and finally cut into 4 μm sections. Firstly, tissue sections were dewaxed with xylene and rehydrated in graded ethanol (100%, 95%, 90%, 80%, 70% and 50%). Secondly, 3% hydrogen peroxide was used to block endogenous peroxidase for 10 mins. Then, antigen retrieval was performed in pressure cooker for 13 minutes with 0.01 M citrate buffer (pH 6.0). Subsequently, ATG12 and BID antibodies were incubated at 4℃ for overnight and second antibody was incubated at room temperature for 1 hour. Finally, the sections were visualized with 3,3′‐diaminobenzidine (DAB). Staining extent was scored from 4 to 0, corresponding to the proportion of positive cells (76‐100%, 26%–75%, 6%–25%, 1%–5%, and 0%). Staining intensity was scored as strong (score = 2), weak (score = 1) or negative (score = 0). A score ranging from 0 to 8 was calculated by multiplying the staining extent and intensity. Final positive degree (negative, 1+, 2+ and 3+) was assigned to each specimen with scores of 0‐1, 2‐3, 4‐5 and 6‐8, respectively.^12^ ATG12 (*Wanlei, Cat#WL03144,1:200*); BID (*Wanlei, Cat#WL01129,1:200*).

### 2.10 Statistical analysis

SPSS23.0 software (IBM) was used for statistical analysis. T test and chi‐square test were used in our study. *P*‐value less than .05 was considered statistically significant.

## 3 RESULTS

### 3.1 Identification of differentially expressed ATGs

**FIGURE 1:**
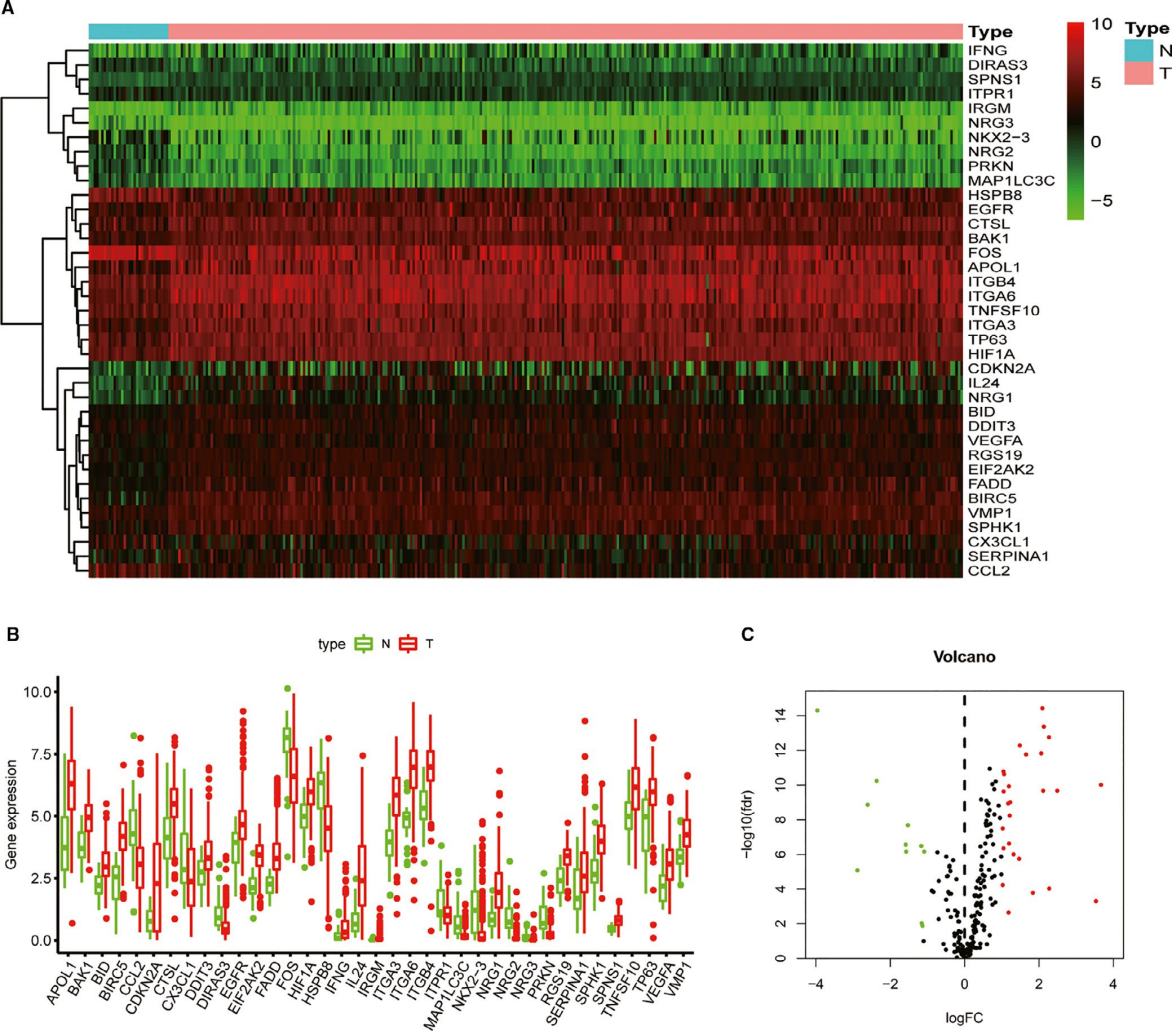
Distributions of autophagy‐related genes (ATGs) in OSCC. (A); The heatmap of 32 normal controls and 317 OSCC samples ATGs expression level. N stands for normal control, while T represents tumour sample. (B); Differentially expressed 37 ATGs in OSCC. N stands for normal control, while T represents tumour sample. (C); The volcano plot of 37 differentially expressed ATGs. Red stands for up‐regulated genes, green represents down‐regulated ATGs and blank means not differentially expressed ATGs

317 OSCC patients and 32 normal controls RNA‐seq and corresponding clinical data were downloaded from TCGA database. 232 ATGs expression level were extracted from the transcriptome data subsequently, and differentially expressed analysis was performed in R software EdgeR package (Figure 1A). With the cut‐off criteria |log_2_FoldChange|>1 and FDR < 0.01 (Figure 1B,C), 11 up‐regulated and 26 down‐regulated ATGs were sorted out. GO enrichment analysis indicated that these genes were mainly located in autophagosome membrane and associated with autophagy (Figure S1A,C). KEGG pathway analysis showed that most of significant ATGs were enriched in apoptosis, platinum drug resistance, ErbB signalling pathway and TNF signalling pathway (Figure S1B,D).

### 3.2 Establishment of cox regression model

**FIGURE 2:**
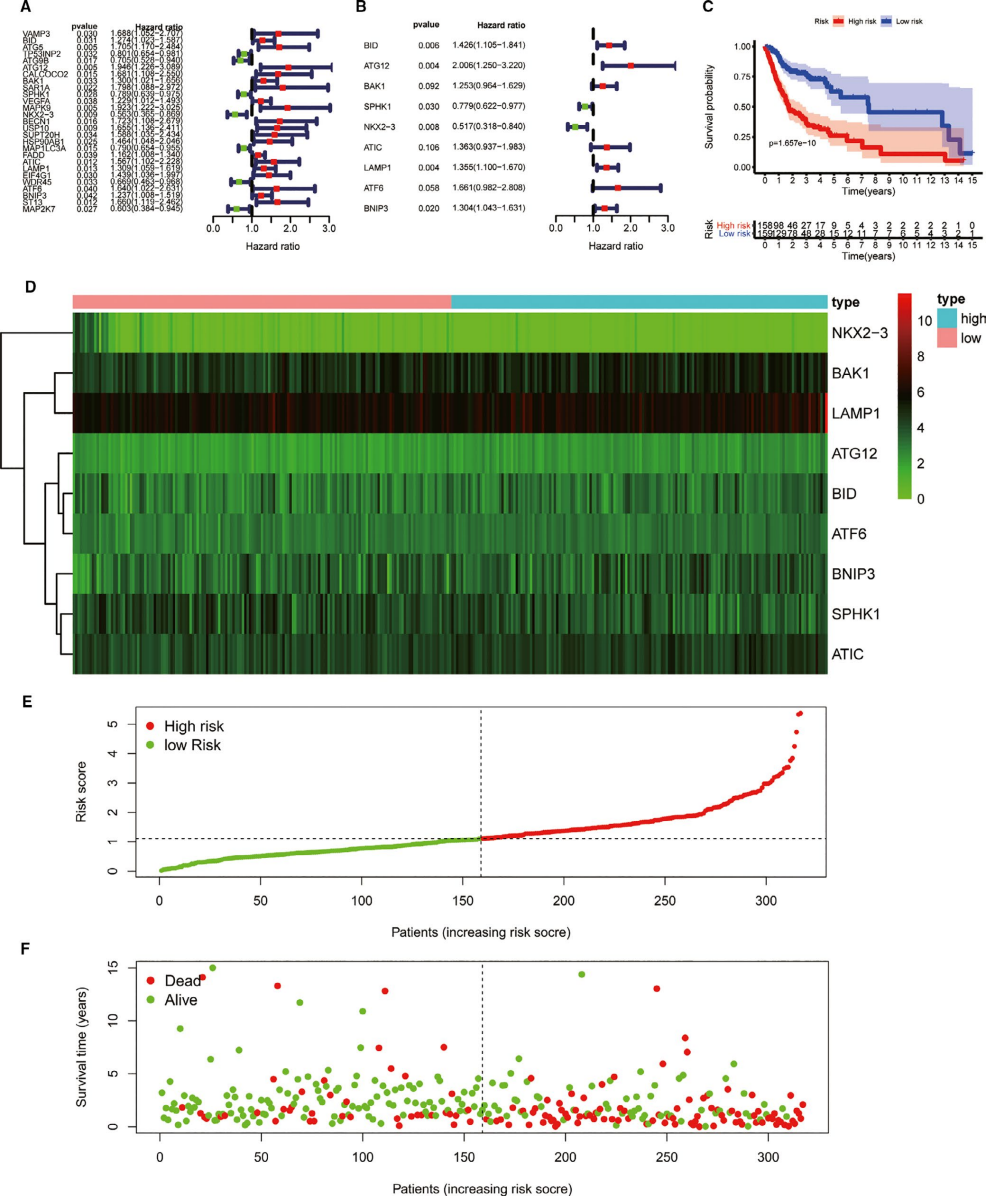
Construction of Cox regression model. (A); Univariate cox regression on basis of all ATGs. (B); All prognostic genes were enrolled in multivariate Cox regression and stepwise Cox regression. (C); Survival analysis was performed according to Cox regression formula. (D); The heatmap of 9 prognostic factors expression profiles. (E, F); Risk score combined with survival time was visualized in R software

According to univariate cox regression analysis, 27 ATGs might be prognostic factors in OSCC (Figure 2A) which then were enrolled in multivariate Cox regression and stepwise Cox regression analysis. Total of 9 variables including BID, ATG12, BAK1, SPHK1, NKX2−3, ATIC, LAMP1, ATF6 and BNIP3 were enrolled in Cox model (Figure 2B). The risk score = (0.35502*BID)+(0.69633*ATG12)+(0.22561*BAK1)‐(0.24922*SPHK1)‐(0.66016*NKX2‐3)+(0.30945*ATIC)+(0.30416*LAMP1)+(0.50726*ATF6) +(0.26573*BNIP3). Subsequently, OSCC patients in TCGA database were divided into high‐risk and low‐risk group according to Cox formula median. Survival analysis indicated that the overall survival rate of high‐risk group was significantly lower than that of the low‐risk group (Figure 2C). Moreover, the expression levels of protective ATGs in the low‐risk group were higher than that of high‐risk group. On the contrary, the expression levels of potential oncogenes were higher in high‐risk group (Figure 2D). In addition, the risk scores combined with survival data were visualized in R software (Figure 2E,F).

### 3.3 Identification of Cox regression model

**FIGURE 3:**
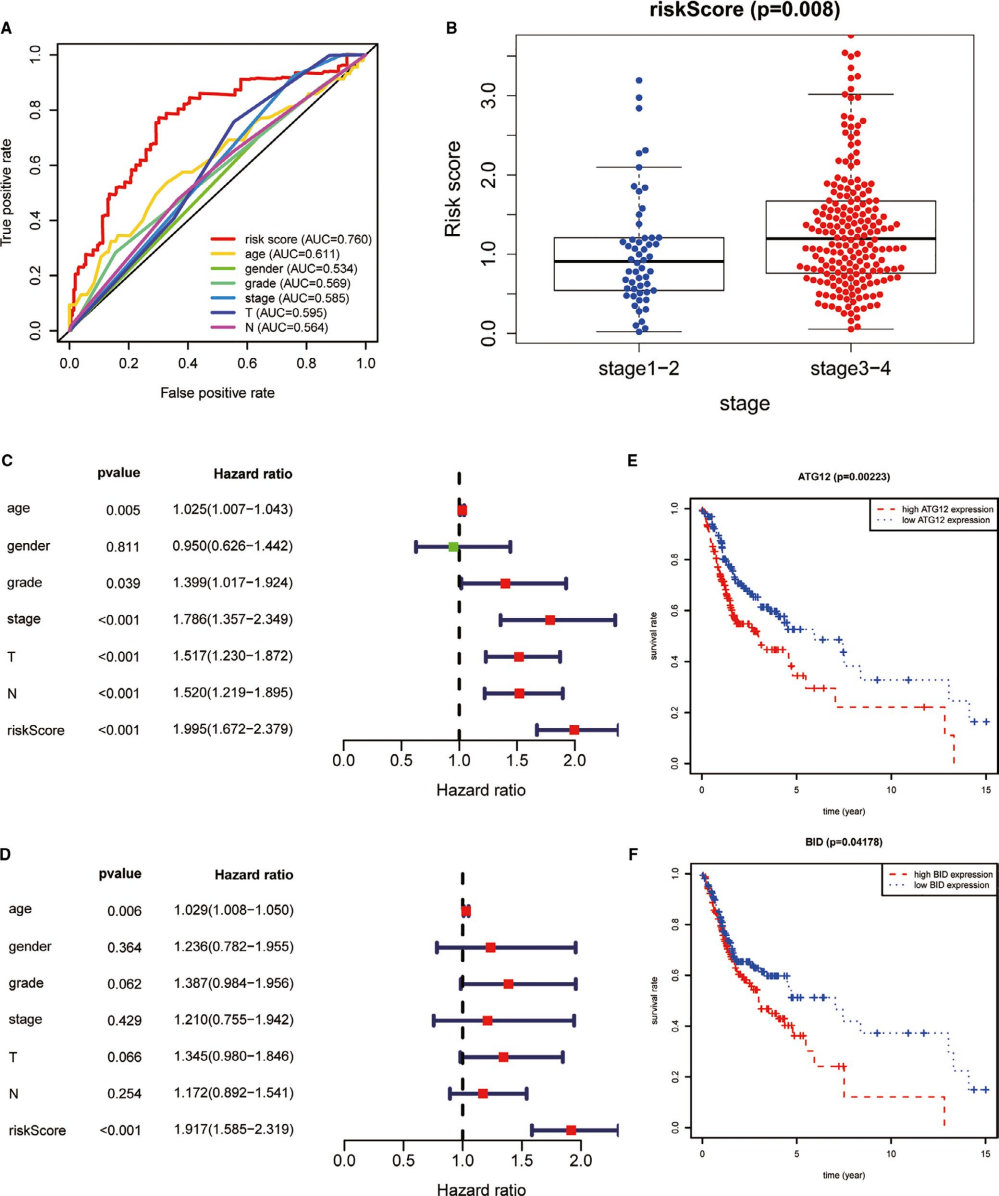
Identification of independent prognostic biomarker. (A); The ROC was plotted according to ATG expression signature, and its area under the curve (AUC) is 0.76. (B); Difference in risk score between early stage (stage 1 + 2) and terminal stage (stage 3 + 4). (C, D); Univariate and multivariate Cox regression analyses reveal that the risk score on basis of ATG expression level can be regarded as an independent prognostic biomarker. (E, F); Survival analysis was performed to analyse these ATGs enrolled in Cox regression model

Firstly, the ROC on basis of cox model was plotted, and its area under the curve (AUC) was 0.76 which was markedly higher than other clinical characteristics (Figure 3A). Furthermore, risk score in early stage was significantly lower than that in advanced stage (Figure 3B) indicating that the risk score on basis of ATGs might realize early diagnosis in OSCC. Moreover, univariate and multivariate Cox regression analysis indicating that the risk score might be regarded as an independent prognostic factor (Figure 3C,D). Moreover, ATG12 and BID were identified as 2 independent autophagy‐related biomarkers according to univariate, multivariate Cox regression analysis (Figure 2A,B) and survival analysis (Figure 3E,F). Unfortunately, ATG12 was not the differentially expressed ATGs. However, integrated analysis indicated that ATG12 might play a carcinogenic role in OSCC. The reasons why ATG12 was not differentially expressed ATGs in OSCC might be that the cut‐off criteria was too high. Therefore, statistical analysis between high and low ATG12 expression group was performed, and the result showed that ATG12 was significantly up‐regulated in tumour samples (Figure S2A).

### 3.4 Survival analysis

**FIGURE 4:**
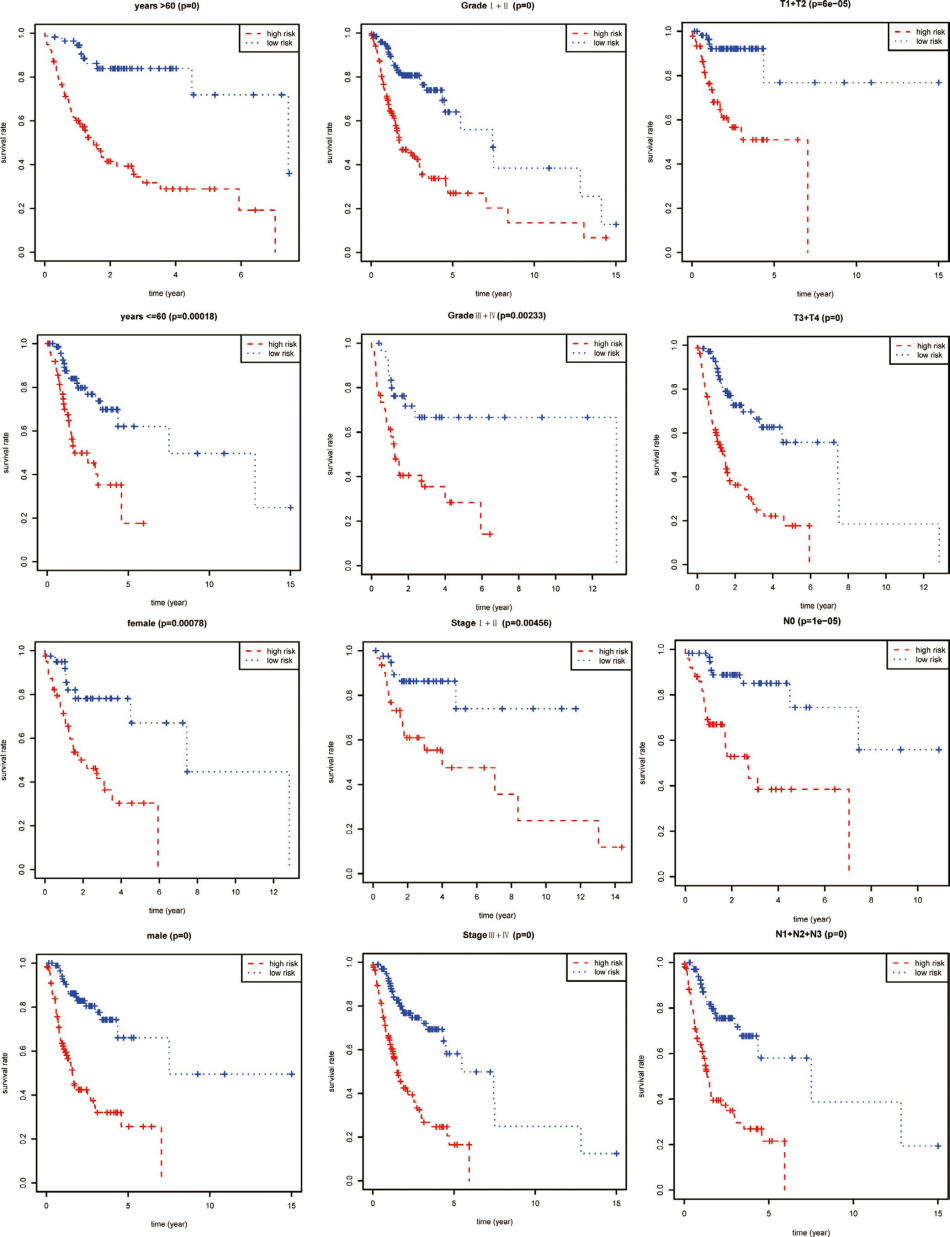
Stratification analysis combined with survival analysis was used to identify the accuracy of the ATG expression signature

The prognostic value of the risk score for different clinicopathological parameters including age, gender, T and N in TNM system, grade and stage was further investigated. M stage in TNM system was excluded because of numerous data missing. Survival analysis combined with stratification analysis including age, gender, T, N, grade and stage demonstrated that low‐risk group had significantly higher overall survival rate than high‐risk group (Figure 4).

### 3.5 Identification of potential independent prognostic biomarkers

**FIGURE 5:**
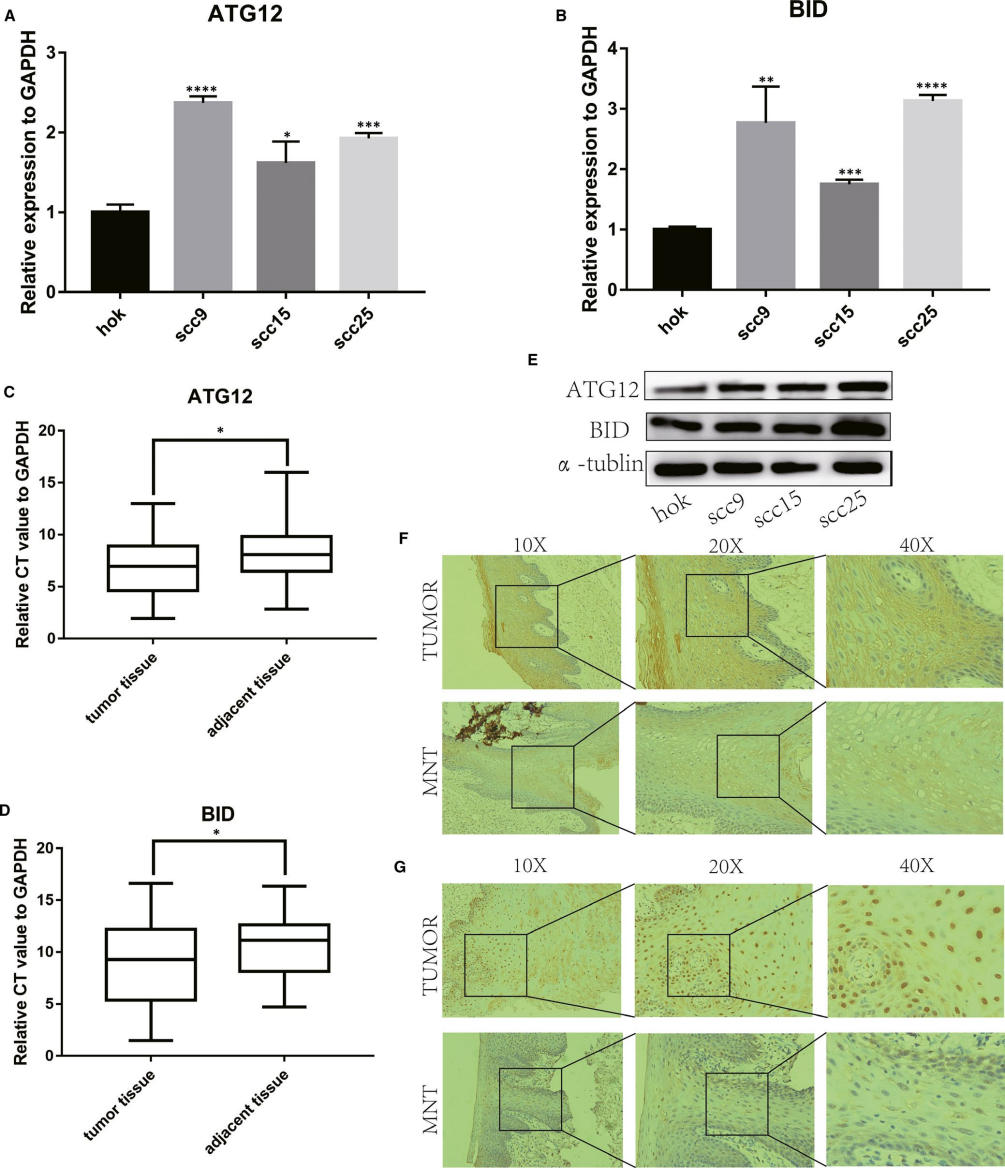
Validation of autophagy‐related biomarkers. (A); ATG12 was up‐regulation in OSCC cell lines (scc9 *P* < .0001, scc15 *P* = .0195, scc25 *P* = .0002). (B); BID expression level in OSCC cell lines (scc9 *P* = .0071, scc15 *P* = .0001, scc25 *P* < .0001). (C, D); Total RNA of ATG12 (*P* = .0139) and BID (*P* = .0141) in 50 OSCC patients and adjacent tissues. (E); The protein level of ATG12 and BID in hok, scc9, scc15 and scc25 cell lines. (F); This represents results of ATG12 immunohistochemistry. TUMOUR stands for OSCC tissue, while MNT means matched normal tissue. (G); This represents results of BID immunohistochemistry. TUMOUR stands for OSCC tissue, while MNT means matched normal tissue

Comprehensive bioinformatics analysis indicated that ATG12 and BID might be associated with the overall survival and played a carcinogenic role in OSCC. Univariate and multivariate Cox regression analysis showed that ATG12 and BID might be selected as potential independent prognostic biomarkers in our study. Therefore, the expression levels of ATG12 and BID were validated in OSCC cell lines and tissues by qRT‐PCR, Western blot and immunohistochemistry. Our results revealed that expression of ATG12 and BID at mRNA level were up‐regulation in OSCC cell lines (Figure 5A,B) and in 50 OSCC patients than MNTs (Figure 5C,D), which were similar with the results in TCGA database. Moreover, ATG12 and BID were also up‐regulated in OSCC cell lines and tissues by Western blot assay and IHC staining, respectively (Figure 5E, Figure 5F,G, Figure S2B,C). However, the mRNA levels of ATG12 and BID had no significant correlation with the clinical parameters (Table 1, Table 2).

**TABLE 1:** Correlation between ATG12 expression and clinical parameters in OSCC patients (n = 50)

Parameters	n	ATG12		*P*‐value
		High	Low	
		expression	expression	
Age(years)				
> = 60	31	14	17	.5607
<60	19	11	8	
Gender				
male	28	17	11	.3926
female	22	10	12	
Stage				
Ⅰ+Ⅱ	32	20	12	.5514
Ⅲ+Ⅳ	18	9	9	
T classification				
T1+T2	35	15	20	.3580
T3+T4	15	9	6	
N classification				
N0+N1	37	19	18	.1906
N2+N3	13	10	3	
Distant metastasis				
M0	40	28	12	0.7067
M1	10	6	4	

**TABLE 2:** Correlation between BID expression and clinical parameters in OSCC patients (n = 50)

Parameters	n	BID		*P* value
		High	Low	
		expression	expression	
Age(years)				
> = 60	29	12	17	.5675
<60	21	11	10	
Gender				
male	32	19	13	.5647
female	18	9	9	
Stage				
Ⅰ+Ⅱ	33	21	12	.3659
Ⅲ+Ⅳ	17	8	9	
T classification				
T1+T2	27	15	12	.2410
T3+T4	23	17	6	
N classification				
N0+N1	31	18	13	.7742
N2+N3	19	10	9	
Distant metastasis				
M0	36	20	16	.7517
M1	14	9	5	

## 4 DISCUSSION

Despite that advancements in surgery, radiation and chemotherapy for the treatment of OSCC, the exact pathogenesis of OSCC is not clear and the 5‐year overall survival is still not satisfied.^13^ Hence, it is crucial to explore the effective biomarkers and therapeutic targets to improve the overall survival of OSCC. Recently, increasing evidences revealed that autophagy might play a significant role in OSCC initiation and progression.^10^, ^14^, ^15^ Therefore, our study aimed to analyse the ATGs by bioinformatics methods and then filter the potential therapeutic targets for OSCC.

A total of 232 ATGs were enrolled in this study. 37 genes were differentially expressed in OSCC which were associated with autophagy and apoptosis by GO enrichment analysis, indicating that these differentially expressed ATGs were relevant to cancer progression.^16^, ^17^ The KEGG pathway analysis revealed that these ATGs were mainly involved in 3 pathways (Figure S1B), of which platinum drug resistance pathway might play an important role in OSCC treatment strategies ^18^ and ErbB signalling pathway was relevant to to head and neck squamous cell carcinoma (HNSCC) treatment,^19^ indicating that these ATGs might provide potential therapeutic targets for OSCC. In fact, some ATGs were identified as potential biomarkers for OSCC. Tang JY et al^20^ indicated that ATG5 might be an indicator of overall survival and disease recurrence in OSCC and the high ATG5 expression of was related to high tumour grade, advanced clinical stage, large tumour size and lymph node metastasis of OSCC, indicating that autophagy play a crucial role in OSCC tumorigenesis and progression. However, the prognostic value of other ATGs in OSCC was unknown.

In the present study, we established a risk model according to 27 prognostic ATGs to predict the overall survival of OSCC. Ultimately, 9 ATGs were selected in Cox regression model and the risk score based on ATG expression level could predict the overall survival accurately. Meanwhile, univariate, multivariate Cox regression and survival analysis combined with stratification analysis indicated that the risk score could be regarded as an independent prognostic factor and distinguish early stage from advanced OSCC, which might be helpful for OSCC early diagnosis and prediction. Furthermore, ATG12 was identified as a potential prognostic biomarker in OSCC which is the human homolog of a yeast protein involved in autophagy.^21^ An increasing number of studies indicated that ATG12 induced autophagy in various human cancers.^22^, ^23^ Qing‐Hua Cao et al^24^ also showed that up‐regulation of ATG12 was correlated with advanced TNM stage and clinical stage in gastric cancer. In head and neck squamous cell carcinoma (HNSC), study showed that high expression level of ATG12 significantly increased the death risk after adjusting for age, gender, clinical stage, smoking and drinking status.^25^ Our study indicated that ATG12 was not the differentially expressed ATGs with the cut‐off criteria |log_2_ (fold change [FC])|>1.0 and FDR (adjusted *P*‐value)<.01 in OSCC. However, statistic analyses showed that ATG12 was up‐regulated in TCGA OSCC samples, OSCC cell lines and tissues. Moreover, univariate, multivariate Cox regression analysis and survival analysis demonstrated that ATG12 might be an effective biomarker and therapeutic target in OSCC. BID, another identified biomarker via bioinformatics analysis in OSCC, locates on chromosome 22q11.21 and encodes a protein associated with apoptosis, which is heterodimerized with apoptotic activator BAX or negative apoptotic regulator BCL_2_. Recently, BID was identified as independent prognostic variables in colon cancer.^26^ Bangbei Wan et al^27^ also demonstrated that BID was a diagnostic biomarker in clear cell renal cell carcinoma (ccRCC). In OSCC, BID might play a carcinogenic role in OSCC progression and be an effective biomarker. Our study showed that overexpression of ATG12 and BID in OSCC tissues had no significant relationship with clinical parameters and prognosis, which might be due to inadequate sample sizes. Moreover, other prognostic ATGs were also related to the pathogenesis of various human cancers. BAK1 play a significant role in ABT‐737‐induced dissociation of the BCL2‐BECN1 complex and autophagy.^28^ SHPK1 promotes the autophagy‐linked lysosomal degradation of CDH1/E‐cadherin and then induces epithelial–mesenchymal transition in hepatoma cells, indicating that the blockage of SPHK1 activity to attenuate autophagy may be a promising strategy for the prevention and treatment of hepatocellular carcinoma.^29^ In addition, LAMP1, ATIC was also associated with various cancers in a autophagy‐dependent manner.^30^, ^31^ Ney PA et al^32^ demonstrated that BNIP3 was associated with mitochondrial autophagy. However, there are fewer studies indicated that these ATGs were related to the development and progression of OSCC in a autophagy manner. These ATGs should be further investigated to explore the roles in OSCC in an autophagy manner.

In summary, we analysed ATGs comprehensively and identified that these differentially expressed ATGs were relevant to OSCC initiation and progression significantly. Furthermore, a 9 ATG gene signature was successfully constructed which was positively associated with overall survival of OSCC. The risk formula might provide potential therapeutic targets for OSCC early diagnosis and treatment. However, there are some limitations in our study. First, the sample sizes in TCGA database and OSCC specimens are markedly inadequate. In addition, the mechanism of ATG12 and BID tumorigenic role in OSCC should be further investigated.

## 5 CONCLUSION

This study successfully constructed a risk model to predict the prognosis of patients with OSCC through comprehensively analysing ATGs, and the risk score might be an independent prognostic biomarker in OSCC. Moreover, ATG12 and BID were also identified as two potential independent prognostic biomarkers for OSCC diagnosis and treatment.

## CONFLICT OF INTEREST

The authors declare that they have no conflict of interest.

## AUTHOR CONTRIBUTIONS

**Guang‐zhao Huang:** Data curation (lead); Formal analysis (lead); Methodology (lead). **Zhi‐yun Lu:** Investigation (lead); Software (lead); Validation (lead). **Yu Rao:** Writing‐original draft (lead). **Hai Gao:** Supervision (equal); Writing‐review & editing (equal). **Xiao‐zhi Lv:** Funding acquisition (lead); Project administration (lead); Supervision (equal); Writing‐review & editing (supporting).

## ETHICAL APPROVAL

The Nanfang Hospital ethics committee (AF/SC‐09/03.2) approved this study.

## CONSENT FOR PUBLICATION

All co‐authors were consent for publication.

## Supporting information

Figure S1

Figure S2

## Data Availability

All data for this study are available from corresponding authors if required.
