# Supplementary figures and images for: Screening and identification of autophagy‐related biomarkers for oral squamous cell carcinoma (OSCC) via integrated bioinformatics analysis
Source: J Cell Mol Med. 2021 Apr 9;25(9):4444–54. doi: 10.1111/jcmm.16512 (PMC8093968; doi:10.1111/jcmm.16512)

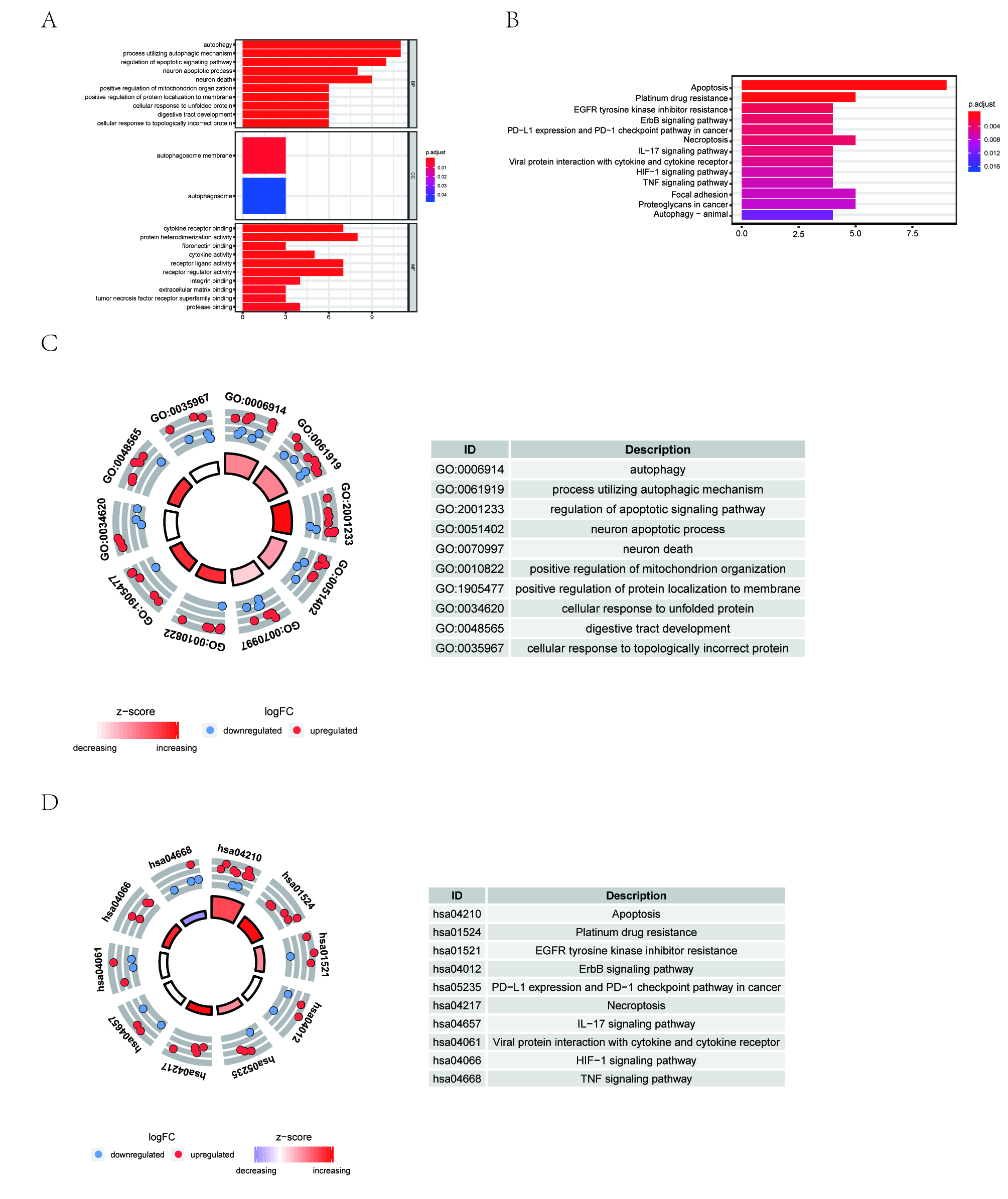

Supplement: Supplementary file 1 — Figure S1 [file JCMM-25-4444-s002.tif]

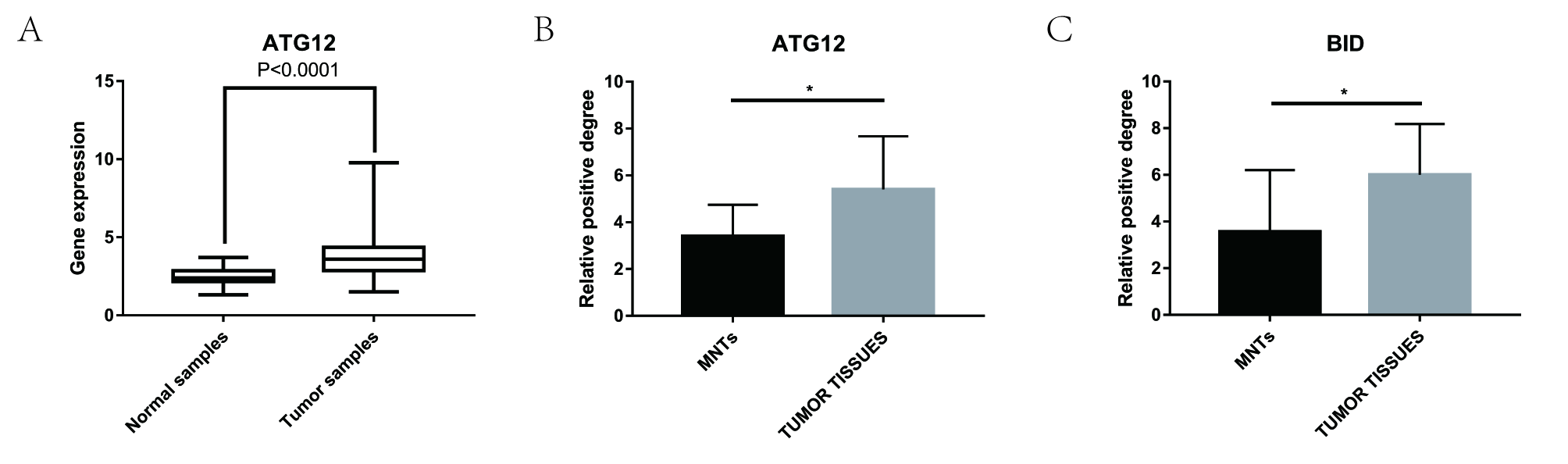

Supplement: Supplementary file 2 — Figure S2 [file JCMM-25-4444-s001.tif]
